# Physical and physiological profile of U18, U19, U21 and senior elite netball players

**DOI:** 10.17159/2078-516X/2020/v32i1a6545

**Published:** 2020-01-01

**Authors:** CJ Sinclair, F Coetzee, R Schall

**Affiliations:** 1 Department of Exercise and Sports Sciences, Faculty of Health Sciences, University of the Free State, Bloemfontein, South Africa; 2 Department of Mathematical Statistics and Actuarial Science, Faculty of Natural and Agricultural Sciences, University of the Free State, Bloemfontein, South Africa

**Keywords:** sport, fitness tests, body composition, performance tests, fitness norms

## Abstract

**Background:**

Physical and physiological profile data for elite netball players in South Africa and internationally are limited but are necessary for conditioning programme information.

**Objective:**

To determine the physical and physiological profiles of U18, U19, U21 and senior level elite netball players at provincial level in the Free State, South Africa. The information provided is by age group and playing position. The fitness of the players for South African and New Zealand netball is also given using the fitness normative data (norms).

**Methods:**

This cross-sectional, descriptive study consisted of 77 elite South African netball players. Anthropometric measurements were taken according to international standards. Fitness tests included the Star Execution Balance Test, standing broad jump, double- and single-leg vertical jump, Yo-Yo Intermittent Recovery Level 1(IR1) test, sprints over 5, 10 and 40 m, horizontal pull-ups and press-ups, the prone bridge test and anaerobic Octorepeater tests with 10 m and 20 m repeated shuttle sprints. In keeping with the descriptive nature of the study, descriptive statistics were calculated for numerical data by age group and playing position.

**Results:**

Players generally did not meet the accepted fitness standards in the following areas: press-ups (all age groups), horizontal pull-ups (senior and U21), standing broad jump (senior and U21), vertical squat jump (senior and U21), 5 m and 10 m sprints (senior and U21); anaerobic Octorepeater (senior players), and the aerobic Yo-Yo IR1 test (all age groups).

**Conclusion:**

Strength and conditioning coaches should develop training programmes to address fitness areas where players do not meet the international standards.

Netball is a team sport with a high level of participation in the Commonwealth, in particular in the United Kingdom, Australia, South Africa and New Zealand (NZ).^[1]^ In South Africa (SA), netball is played in schools, clubs and at provincial level, with approximately half a million players in schools and 9 700 adult players.^[2]^ Netball is a popular women’ s sport, and in South Africa it receives government support to enhance talent identification and sport performance.^[3]^

Limited research has been done internationally that profiles the physical characteristics and fitness levels of netball players. No research describing the physical and physiological profiles of U18, U19, U21 and senior elite netball players in SA has been previously conducted.^[4,5]^ Therefore, this is the objective of the present study, and the data could provide information for coaches, players and netball organisations with regard to sports progression and talent identification.^[9]^

Previous research^[10]^ has indicated that more comprehensive studies are required to ensure that netball players undergo relevant conditioning and technique training to meet the specific demands of the sport. Various fitness tests can be performed to determine a netball player’s physiological profile.^[6–9]^ Such tests will identify the player’ s strengths and weaknesses, providing strength and conditioning coaches with information on the effectiveness of their training methods. The significance of this study is that the results may serve to validate current physical fitness assessments or indicate the need for different assessment and training methods for netball players.

The authors studied players from the Free State (FS) High School’s U18A league and from the FS U19, FS U21 and FS senior netball teams subjecting them to a comprehensive battery of fitness tests. A secondary objective was to compare the fitness profiles of players in this study with fitness normative data for specifically SA and NZ netball.

## Methods

### Participants

This cross-sectional, descriptive study included a total of 96 participants of which 77 completed all the tests. Nineteen participants were excluded from the study due to injuries at the time of fitness testing. All participants were registered members of one of the following teams: FS High School U18A league, FS U19 and FS U21 teams, and FS senior team. Approval for the study was obtained from the Health Sciences Research Ethics Committee, UFS (UFS-HSD2017/0014). Before testing, each participant provided an informed consent form to the researchers and received an information document which provided the details of the research study.

### Testing procedures

All teams were tested at the beginning of the 2017/2018 netball season at the Exercise and Sport Science Centre of the University of the Free State (UFS) in Bloemfontein, South Africa. Tests took place in the mid-afternoon over a two-day period. A logical sequence of tests was compiled to determine the correct order the tests should follow and that the necessary duration of rest periods between tests was ensured for test reliability.^[11]^ The following measurements and tests were performed on the first day from non-fatiguing tests:

#### Anthropometric measurements

Anthropometric measurements according to the International Standards for Anthropometric Assessment (ISAA);^[12]^

- body mass and stature
- the sum of six skinfolds (triceps, subscapular, suprailliac, abdominal, supraspinale, thigh and calf).

#### Star Execution Balance Test (SEBT)

The Star Execution Balance Test (SEBT) which determined the balance and postural control in a dynamic test that is functional in netball.^[1]^

During this test, the participant stands with their hands on their iliac crest centred in a “star sign”. The participant is then instructed to reach out with one foot while balancing on the opposite leg. Each participant is asked to reach out in eight different directions at 45° increments from the centre of the “star sign”. The participant has to reach to the furthest possible point with the distal part of their foot while maintaining their balance, and to return to the start position after each reach position. The distance reached is recorded on the measuring tape that is attached to the star sign. The test is performed on both legs.^[13]^

#### Standing broad jump and double- and single-leg vertical jump

The standing broad jump test of explosive horizontal leg power and double- and single-leg vertical squat jump tests of vertical explosive power which determines the participant’s explosive horizontal leg power. The participant was required to stand behind a marked line with feet shoulder width apart on the ground. The participant had to stand without swaying or rocking their feet but with knees bent, and only swinging their arms. They then had to perform a two-foot forward drive take-off, landing with both feet on the ground simultaneously. The maximum distance achieved via the best of three trials was recorded.^[8]^

Before the double- and single-leg vertical squat jump test, the participant was instructed to stand next to the Vertec vertical jump tester and reaching up, touch the highest vane possible with one hand while maintaining both feet firmly on the ground. This height was recorded as the participant’s standing reach height. Participants performed the best of three trials with sufficient rest between them. The maximal vertical jump was determined as the difference between the maximum height jumped and standing reach height.^[8]^ The reliability and intraclass correlation coefficient (ICC) is excellent for the double-leg vertical jump (0.94) and coefficient of variation (CV) 3.3 cm. The single-leg vertical jump has also an excellent reliability of ICC of 0.96 and 0.91 for the right and left legs respectively, with CV of 4.2 cm.^[14]^

#### Yo-Yo Intermittent Recovery Level 1(IR1) test

The Yo-Yo IR1 Aerobic Test evaluates aerobic fitness over a period of time, which is according to the level deducted from the cd playing. Cones marked out three lines: 5 m (recovery), 0 m (start line) and 20 m (turn line). The participant started on or behind the start line (0 m) and began running 20 m when instructed to by the recorded audio. The participant then turned and returned to the starting point. The participant must then complete the active recovery period (5 s) during which they must walk or jog around the other (5 m) cone and return to the starting point. A warning was given when the participant did not complete a successful out and back shuttle and recovery shuttle in the allocated time. The participant was then told to stop the test. A participant’s final Yo-Yo score was the last successful shuttle completed.^[8}^

The following tests were performed on the second day:

#### Horizontal pull-ups, press-ups and the prone bridge test

Horizontal pull-up and press-up tests to evaluate pulling, pushing strength and endurance, and the prone bridge (plank) to test core endurance.

A weight lifting bar was placed in a squat or power rack. The participant’s arms had to be fully extended, with their body just off the ground. The participant gripped the bar, which was slightly wider than shoulder width, by means of an overhand grasp. Their feet were flat on the floor, with knees bent at a 90° angle. Their hips were then lifted so that their body was straight and their arms fully extended. They then pulled their body towards the bar until their chest touched it (nipple line aligned with bar). They then lowered themselves back down until their arms were again fully extended. The participant continued doing as many repetitions as possible until they could no longer touch the bar. The examiner recorded the total amount of correct repetitions completed.^[8]^

The press-up evaluates the participant’s upper body’s pushing and endurance strength. The start was in the plank position with their hands and knees off the ground. The examiner then placed a closed fist in line with the participant’s chest. They then lowered their body so that it was in line with the fist and thereafter pushed up again until their arms were once more fully extended. The total number of correct repetitions were counted.^[8]^

As previously mentioned, the prone bridge (plank) evaluates the participant’s core strength and endurance. The participant started in the plank position, with elbows on the ground, feet approximately hip distance apart, knees off the ground and the body straight, with no arching of the back. The head and neck faces towards the ground, keeping the whole body aligned. The participant was required to maintain the plank position for as long as possible, maintaining normal breathing. Once the participant can no longer keep their body straight, i.e. they start to excessively arch or curl their back, the test is stopped. The total time that the participant maintained a correct plank position was recorded.^[8]^

#### Sprints

Sprints over 5, 10 and 40 metres (m) respectively to test acceleration and speed.

The aim of this test was to determine the acceleration and rapidity of each participant in sprinting. Electronic timing lights were placed at the start at distances of 5, 10 and 40 m to ensure that there was enough space past the 40 m marker for participants to decelerate and stop. Each participant started from a stationary position with one foot behind the start line, ensuring there was no rocking or swaying prior to the start. An adequate warm-up was provided (5 minutes light jog, 10 minutes dynamic stretches) before the test to minimise risk of injury. Each participant had to complete three trials with approximately two–three minutes of rest between attempts, and the best score was used.^[8]^

#### Octorepeater tests

The Octorepeater anaerobic fitness test, consisting of 10 m and 20 m repeated shuttle sprints, mimics the game of netball.

This test assesses the player’s ability to perform repeated maximal sprints that incorporate a change of direction. Each participant completes four sets of 2 x 20 m and four sets of 4 x 10 m sprints respectively, alternating between the 2 x 20 m and 4 x 10 m sprints, with 25-second rest intervals between each 2 x 20 m and 4 x 10 m set. The participant first completes the 2 x 20 m followed by the 4 x 10 m sprint. The fatigue index is then calculated as 100·(Average time - Best time)/(Best time), where “Average time” is the average time achieved in the four sets of 2 x 20 m and 4 x 10 m sprints, and “best time” is the sum of the best times achieved in the four sets of 2 x 20 m and 4 x 10 m sprints, respectively.^[8]^

### Statistical analysis

In keeping with the descriptive nature of the study, descriptive statistics were calculated for numerical data for all players in the sample. Descriptive statistics were also calculated by age group (U18, U19, U21 and senior players) and playing position. Potential differences between the age groups and playing positions were tested using one-way analysis of variance, and the associated p-value from the F-test was determined in Tables 1, 2 and 3. Because of the relatively large number of tests performed and the resulting multiplicity issues, these p-values should be interpreted descriptively. For the same reason, no post-hoc tests were conducted. Data analysis was performed using SAS Version 9.4.^[15]^

## Results

### Anthropometric measurements

The mean body weight of U18, U 21 and senior players was 66.9 kg, 70.4 kg and 75.3 kg, respectively, suggesting a trend that body weight increases with age (Table 1). However, the mean weight of the U19 group was 76.7 kg, which was inconsistent with the pattern, although this could have been due to the small size (n=5) of the U19 group. Similarly, Table 1 shows a trend that height increases with age. The differences in weight (p=0.046) and height (p=0.011) between the age groups were statistically significant at the conventional significance level of 0.05. However, the body mass index (BMI) p=0.674) and fat percentage (p=0.078) did not differ significantly between age groups.

Body weight, height, body fat percentage and BMI varied according to overall playing position. The goal shooter (GS) had the highest mean body weight of 80.1 kg, followed by the goalkeeper (GK) 77.7 kg and goal attacker (GA) 74.2kg. The GS is the tallest player 181.2 cm compared to the GK and GA that are each the same height of 175 cm. Overall, the differences in weight and height between playing positions were statistically significant (both p<0.0001), while BMI (p=0.416) and fat percentage (p=0.381) did not differ significantly.

Tables 2, 3 and 4 represent the results of the fitness tests that formed part of the profiling of physiological data of the netball players and the percentage of players that were compliant with the SA and NZ standards^[8,19]^ (Table 4).

### Muscular strength endurance and explosive power tests

The variability in the muscular strength endurance and explosive power tests (Table 2) was generally high. The coefficient of variation (mean divided by standard deviation (SD)) of these measurements was generally 30% or higher. None of the prone bridge, press-up, horizontal pull-up, double- or single-leg vertical squat jumps differed significantly between age groups (Table 2). Of the explosive power tests, only the standing broad jump differed significantly (p=0.033) between age groups, with the two younger age groups achieving slightly higher values, although these small differences of 4–7 cm did not appear to be of practical relevance.

In all these tests, only the mean number of press-ups differed significantly (p=0.032) between playing positions (which could have been due to the low mean for the GA). However, the mean differences of approximately one press-up again seemed to be of small practical relevance. With regard to explosive power, only the single-leg vertical squat jump differed significantly between playing positions (p=0.044).

Except in the case of the press-up, the majority of participants (80% or more) in the two younger age groups met the minimum standards set by SA and NZ netball for the respective age groups (Table 4).^[8,19]^ By contrast, notably fewer of the senior and U21 players met the standards for these tests – generally below 50%.

### Speed, aerobic and anaerobic tests

Of all of speed, aerobic and anaerobic tests (Table 3), only the Yo-Yo IR1 test showed statistically significant differences (p=0.020) between the age groups, suggesting a trend for higher mean values as age increases. Differences in the fatigue index between age groups were borderline insignificant (p=0.060), with the senior players (mean of 8.5%) exhibiting higher values than the other age groups (mean between 3.8% and 5.8%). None of these tests showed significant differences between playing positions.

Only a small number of players in all the age groups met the minimum standards for the 5 m and 10 m sprints and the Yo-Yo IR1 test. By contrast, most players in the younger age groups met the standard for the fatigue index, compared to only 45.5% of senior players.

## Discussion

Comparative data on the physical and physiological profiles of U18, U19, U21 and senior elite netball players are not available for SA, and only a limited number of international studies have been conducted.^[7,9,17]^ To these authors’ knowledge, this is the first study to investigate and statistically compare the physical and physiological profiles of the various age groups from U18 to senior level of elite netball players in SA.

The overall mean body weight of netball players in this study was 69.5 kg, which was similar to a study investigating female U19 players that reported a mean weight of 69.8 kg.^[17]^ Another study reported a mean weight of 68.8 kg for netball players aged 18–25 years.^[10]^ The overall mean height of players in this study was 170.9 cm, which was similar to a study conducted on junior and elite senior Malaysian netball players (170.8 cm),^[6]^ as well as a study on players with a mean age of 15.4 years and mean height of 170.0 cm.^[18]^ The current study found the overall fat percentage to be 27.8% and the overall BMI to be 23.7 kg/m^2^. This was in contrast to other studies which found netball players to be notably leaner, such as the Malaysian netball players (fat percentage 24.5%),^[6]^ while two other studies reported a mean BMI of 22 kg/m^2^ and 21.9 kg/m^2^,respectively.^[9,17]^ However, the low BMI values in previous research could be attributed to the mean age of players (14^9^ and 15.4^17^ years, respectively) being lower than in this present study.

Here the mean number of press-ups, namely 9.9, was higher than in a study on U19 NZ secondary school netball players who achieved only 7 repetitions.^[18]^ However, the fitness norms for NZ^[8]^ and SA^[19]^ for U17 to senior elite players specify between 15 and 25 repetitions, and overall, only 19.5% of players met those norms. Furthermore, the overall mean number of repetitions of horizontal pull-ups was 12.5 in the current study, whereas fitness norms range from 5 to 15 repetitions for U17 to senior elite players^[8,19]^, with 71.4% of players meeting the norm. Specifically, the findings regarding press-ups therefore, suggest deficits in upper body strength among the players in this present study. Upper body strength is vital for the player to produce enough power to throw the ball with increased speed and power to her teammate. In the current study, the overall mean prone bridge test score was 117.6 s, compared to U19 NZ secondary school players who only achieved 59.4 s,^[18]^ which is notably lower than in this study. Norms for the various countries range between 60 and 120 s,^[8,19]^ which were met by 69% (SA norm) and 82% (NZ norm) of players respectively. These results might suggest that a higher emphasis on core conditioning might be necessary.

In this present study, the overall mean value for the standing broad jump (horizontal explosive power) was 1.75 m, whereas a NZ study reported 1.82 m for U17 and 1.69 m for U19 secondary school players.^[18]^ The norms for NZ and SA range between 1.70 and 2.10 m from U17 to senior elite players,^[8,19]^ and these were met by 79% (SA norm) but only 17% (NZ norm) of players. With regard to vertical explosive power, this study found the overall mean values of 43.9 cm for the double-leg vertical squat jump and 34.8 cm for the single-leg vertical squat jump. Australia’s norms for U17 to senior national team players are 43.9 cm to 46.4 cm.^[16]^ The norms for NZ and SA range from 40 cm to 50 cm for the double-leg vertical squat jump and 30 cm to 40 cm for the single-leg vertical squat jump test for U17 to elite level players,^[8,19]^ with only about two-thirds of SA players meeting the relevant norm. A lack of explosive power can result in a player failing to retain the ball when jumping up or leaping forward to catch an oncoming ball. Additionally, in Table 2 the C had the most (DL 47.2 cm; SL 39.8 cm) explosive power with the double- and single-leg squat jump, while the GK the least (DL 41.7 cm; SL3 2.4 cm). This is similar to results found by Thomas et al.^[20]^ where the C jumped higher than shooters or defenders.

The mean time for the 5 m sprint in this study was 1.27 s overall, which was 0.04 s slower than the mean of 1.23 s reported for a sample of a similar age group in Australia.^[16]^ The norms for NZ and SA range from 1.15 s 1.08 s for U17 to senior level players.^[8,19]^ Overall, only 12% and 5% of players met these norms. In the present study, the overall mean time achieved for the 10 m sprint was 2.15 s, which was notably slower than the norms which range from 1.95 s to 1.85 s^[8]^ for NZ and from 2.00 s to 1.90 s for SA.^[19]^. Accordingly, 10% and 3% of players met these norms. Previous research reported a mean time of 2.05 s for the 10 m sprint for players from U17 to senior level.^[16]^ These results suggest that players in the current study were too slow in the 5 m and 10 m sprints.

The Octorepeater is a test that mimics the demands of netball match play of constant short bursts of speed (sprints) and constant changes in direction. The overall mean time for eight maximal sprints with 25 s intervals in the current study was 83.77 s while the norm for NZ and SA for U17 to senior level players ranges from 85 s to 70 s.^[8,19]^

The fatigue index indicates how quickly the player decreases in speed or fatigues over the eight sprints. It provides an indication of the anaerobic capacity of a player when executing short bursts of speed during match play. The overall mean fatigue index in the current study was 5.9%, compared to NZ and SA norms of between 5% and 8%.^[8,19]^ Only approximately half of the senior netball players in the current study met these norms, while most of junior players did. The overall mean score for the Yo-Yo IR1 test was level 14.6, in comparison with both the NZ and SA (level 15.1 – level 17.5) norms of level 15.1–18.5 and level 15.1–17.5, respectively.^[8,19]^ Only 7% and 4% of players respectively met these norms. Thus the Yo-Yo IRI test results suggest that the aerobic fitness levels of the players in the current study did not meet international norms.

### Practical application

The findings in this study suggest that the study population did not meet, or only partially met, national and international norms in the various fitness areas of the physiological profiling of netball players. Furthermore, this study’s results study show that there are differences in the physical and physiologic characteristics in the different playing positions and age groups of elite netball players. Fitness for netball cannot be determined by a single parameter because the game demands a large number of physical and physiologic capabilities which are presented in this study. Successful play at elite level in netball depends on how individuals knit together into a complete unit. Thus the combination of physical and physiologic characteristics may vary from player to player or in the different positions in the game.

Therefore, based on the results of this study, it is recommended that increased emphasis on the following training modalities should be considered: endurance strength in the upper body; vertical and horizontal explosive power, and explosive speed over short distances. According to Thomas et al.^[18]^ greater lower limb relative strength is required to overcome the inertia of body mass and improve a player’s ability to accelerate and decelerate during certain movements, such as jumping, sprinting and changing in direction, thereby reducing the risk of injury and enhancing performance.

Additionally, high-intensity training modalities, such as repeated short (10 s – 1 min) to long (2–3 min) bouts of high-intensity shuttle running, interval training which improves aerobic endurance, anaerobic capacity, and maximum aerobic speed.^[18]^ Çiçek et al.^[21]^ provided evidence that aerobic training, such as 2 km time trials, will improve forced vital capacity, forced expired volume in one second VO_2_max (ml/kg/min).

## Conclusion

Conditioning coaches can use this information to determine which type of profile is needed for a specific position or age group. Experienced netball coaches can use this information in the process of designing a training programme to maximise the fitness development of netball players.

## Figures and Tables

**Table 1:** Anthropometric measurements of elite netball players according to age group and playing position

Variable	Statistic	Age group				F-test (df=3,73)	Playing position							F-test (df=6,70)	All (n=77)
	
		Senior (n=11)	U21 (n=16)	U19 (n=5)	U18 (n=45)		GS (n=11)	GA (n=4)	WA (n=15)	C (n=15)	WD (n=14)	GD (n=7)	GK (n=11)		
**Age (yr)**	**Mean**	20.9	19.7	19.0	16.6		18.2	18.5	17.8	18.0	18.5	17.4	18.0		18.1
	**SD**	1.6	0.9	0.0	0.7		2.0	3.1	1.5	1.6	2.6	1.5	2.2		2.0
	**Min**	18	18	19	15		16	16	16	15	15	16	15		15
	**Max**	23	21	19	18		22	23	21	21	22	20	22		23

**Mass (kg)**	**Mean**	75.3	70.4	76.7	66.9	F=2.80; p=0.046	80.1	74.2	63.3	64.7	66.8	66.2	77.7	F=6.20; p<0.0001	69.5
	**SD**	10.7	6.7	17.1	10.9		10.6	7.3	6.9	8.00	9.7	8.8	12.0		11.0

**Height (cm)**	**Mean**	176.7	172.7	172.8	168.6	F=3.97; p=0.011	181.2	175.0	165.5	167.6	167.9	170.4	175.0	F=9.00; p<0.0001	170.9
	**SD**	7.8	5.2	5.9	8.5		5.9	5.7	6.8	4.5	8.2	7.9	5.7		8.1

**BMI (kg/m2)**	**Mean**	24.1	23.6	25.2	23.5	F=0.51; p=0.674	24.5	24.3	23.1	23.0	23.8	22.7	25.2	F=1.03; p==0.416	23.7
	**SD**	2.4	2.4	4.1	3.2		4.1	2.5	1.7	2.4	3.6	1.7	3.5		3.0

**Body fat (%)**	**Mean**	28	28.2	32.9	27.1	F=2.36; p=0.078	30.0	29.6	27.3	26.3	27.1	26.4	29.4	F=1.08; p=0.381	27.8
	**SD**	4.4	4.5	6.6	4.7		4.4	3.8	4.8	5.0	4.6	2.1	6.5		4.84

p-value calculated from the F test of one-way analysis of variance (ANOVA).

SD, standard deviation; BMI, body mass index; F, F-statistic; p, P-value; df, degrees of freedom (numerator, denominator); GS, goal shooter; GA, goal attack; WA, wing attack; C, centre; WD, wing defence; GD, goal defence; GK, goal keeper.

**Table 2:** Muscular strength endurance and explosive power tests of U18, U19, U21 and senior elite netball players

Variable	Statistic	Age group				F-test(df=3,73)	Playing position							F-test (df=6,70)	All (n=77)
	
		Senior (n=11)	U21 (n=16)	U19 (n=5)	U18 (n=45)		GS (n=11)	GA (n=4)	WA (n=15)	C (n=15)	WD (n=14)	GD (n=7)	GK (n=11)		
**Prone bridge (s)**	**% SA**	55	37.5	100	80	F=1.43 p=0.240	73	75	60	80	36	86	91	F=0.92 p=0.484	69
	**% NZ**	55	37.5	100	93		73	86	87	93	64	86	91		81.8
	**Mean**	116.0	97.4	105.0	127		109.1	116.0	118.1	123.1	98.3	115.6	144.6		117.6
	**SD**	45.0	36.0	23.3	57.6		65.8	23.3	50.5	38.4	36.3	10.4	75.9		51.1

**Press-up (n)**	**% SA/ NZ**	9	13	0	27	F=0.36 p=0.785	9	0	33	7	14	29	36	F=2.47 p=0.032	20
	**Mean**	8.9	9.8	7.0	10.4		11.7	2.0	12.9	11.1	10.2	10.4	11.7		9.9
	**SD**	6.9	7.3	4.7	8.5		4.9	1.8	9.9	7.8	7.5	6.0	6.0		7.8

**Horizontal pull-up (n)**	**% SA/ NZ**	46	56	80	82	F=0.66 p=0.580	73	75	60	73	57	86	91	F=0.62 p=0.715	71
	**Mean**	12.5	11.1	11.2	13.2		10.9	11.3	12.5	14.6	11.6	12.9	13.0		12.5
	**SD**	4.3	6.5	4.9	5.5		5.0	3.0	5.5	6.7	6.4	4.2	4.8		5.5

**Standing broad jump (m)**	**% SA**	46	81	100	84	F=3.07 p=0.033	46	25	93	87	86	86	91	F=2.07 p=0.068	79
	**% NZ**	9.1	18.8	20.0	17.8		0.0	0.0	13.3	26.7	14.3	28.6	27.3		16.9
	**Mean**	1.83	1.82	1.87	1.89		1.8	1.6	1.8	1.8	1.6	1.8	1.8		1.8
	**SD**	0.2	0.2	0.2	0.2		0.2	0.3	0.3	0.2	0.2	0.2	0.2		0.2

**Double-leg vertical squat jump (cm)**	**% SA/ NZ**	46	63	100	69	F=0.96 p=0.418	54.6	25	60	80	57	86	82	F=1.34 p=0.251	66
	**Mean**	46.4	43.2	45.8	43.3		45.3	42.3	43.9	47.2	41.5	43.9	41.7		43.9
	**SD**	5.3	7.0	5.7	6.2		6.1	4.8	2.5	6.1	6.7	7.1	6.1		6.2

**Single-leg vertical squat jump (cm)**	**% SA/ NZ**	46	25	100	80	F=1.82 p=0.151	55	50	67	73	43	71	91	F=2.30 p=0.044	65
	**Mean**	36.6	33.7	39.4	34.3		36.0	24.3	33.7	39.8	34.0	33.5	32.4		34.8
	**SD**	6.7	4.7	4.0	5.8		5.7	1.5	2.8	5.4	7.2	5.4	5.4		5.8

p-value calculated from the F test of one-way analysis of variance (ANOVA)

%SA, percentage of players compliant with the minimum standards of South Africa; %NZ, percentage of players compliant with the minimum standards of New Zealand; %SA/NZ, percentage of players compliant with the minimum standards of both South Africa and New Zealand; SD, standard deviation; BMI, body mass index; F, F-statistic; p, P-value; df, degrees of freedom (numerator, denominator); GS, goal shooter; GA, goal attack; WA, wing attack; C, centre; WD, wing defence; GD, goal defence; GK, goal keeper.

**Table 3:** Speed, aerobic and anaerobic tests of U18, U19, U21 and senior elite netball players

Variable	Statistic	Age group				F-test (df=3,73)	Playing position							F-test (df=6,70)	All (n=77)
	
		Senior (n=11)	U21 (n=16)	U19 (n=5)	U18 (n=45)		GS (n=11)	GA (n=4)	WA (n=15)	C (n=15)	WD (n=14)	GD (n=7)	GK (n=11)		
**5 m sprint (s)**	**% SA**	0	0	0	20	F=0.30 p=0.827	9	25	20	7	14	0	9	F=1.15 p=0.343	12
	**% NZ**	0	0	0	9		9	0	7	7	0	0	9		5
	**Mean**	1.3	1.3	1.3	1.3		1.3	1.3	1.2	1.2	1.3	1.3	1.3		1.3
	**SD**	0.1	0.1	0.1	0.1		0.1	0.1	0.1	0.1	0.1	0.1	0.1		0.1

**10 m sprint (s)**	**% SA**	0	0	0	18	F=0.20 p=0.898	9	0	27	13	0	14	0	F=1.78 p=0.117	10
	**% NZ**	0	0	0	4		9	0	7	0	0	0	0		3
	**Mean**	2.1	2.2	2.2	2.1		2.2	2.2	2.1	2.1	2.2	2.1	2.2		2.2
	**SD**	0.1	0.1	0.1	0.2		0.2	0.1	0.1	0.1	0.1	0.1	0.1		0.1

**40 m sprint (s)**	**% NZ**	100	100	100	95.6	F=0.18 p=0.908	91	100	100	100	100	100	91	F=1.26 p=0.287	97
	**Mean**	6.5	6.5	6.7	6.5		6.8	6.6	6.6	6.3	6.6	6.5	6.6		6.5
	**SD**	0.4	0.4	0.3	0.6		0.9	0.4	0.4	0.2	0.4	0.5	0.5		0.5

**Total time (s)**	**%SA/NZ**	0	0	80	56	F=0.37 p=0.774	9	0	13	18	11	2	11	F=1.62 p=0.155	38
	**Mean**	85.22	84.38	82.43	83.35		86.78	88.16	84.21	81.32	82.91	86.06	81.54		83.77
	**SD**	6.60	3.74	2.63	7.29		9.50	8.53	6.13	4.35	5.78	5.45	4.01		6.34

**Fatigue index (%)**	**%SA/NZ**	46	100	100	84	F=2.57 p=0.060	82	75	80	93	79	71	99	F=1.11 p=0.365	83
	**Mean**	8.5	4.9	3.8	5.8		7.0	4.7	7.6	4.4	5.1	5.8	6.1		5.9
	**SD**	5.4	1.5	1.3	4.2		4.0	3.0	6.1	1.9	2.0	3.8	4.4		4.0

**Yo-Yo (level)**	**% SA**	0	13	0	7	F=3.51 p=0.0195	9	0	0	0	0	29	18	F=1.39 p=0.2300	7
	**% NZ**	0	0	0	7		9	0	0	0	0	14	9		4
	**Mean**	15.2	15.00	14.8	14.3		14.1	14.5	14.5	15.2	14.4	15.00	14.6		14.6
	**SD**	0.6	0.9	0.5	1.1		1.2	1.6	1.2	0.7	1.0	1.3	0.7		1.1

p-value calculated from the F test of one-way analysis of variance (ANOVA).

%SA, percentage of players compliant with the minimum standards of South Africa; %NZ, percentage of players compliant with the minimum standards of New Zealand; %SA/NZ, percentage of players compliant with the minimum standards of both South Africa and New Zealand; SD, standard deviation; BMI, body mass index; F, F-statistic; p, P-value; df, degrees of freedom (numerator, denominator); GS, goal shooter; GA, goal attack; WA, wing attack; C, centre; WD, wing defence; GD, goal defence; GK, goal keeper.

**Table 4:** Minimum fitness standards of South Africa and New Zealand elite netball players

Test	Country	Senior			U21			U19 & U18		

		GK/GS	GA/GD	WA/WD/C	GK/GS	GA/GD	WA//WD/C	GK/GS	GA/GD	WA/WD/C
**Prone bridge (s)**	**SA**	90	105	120	90	105	120	60	60	90
	**NZ**	120	120	120	90	90	90	60	60	60

**Press-ups (n)**	**SA/NZ**	20	25	25	15	20	20	15	15	15

**Horizontal pull-up (n)**	**SA/NZ**	15	15	15	10	10	15	5–8	8–10	10

**Standing broad jump (m)**	**SA**	1.90	1.90	1.90	1.70	1.70	1.70	1.50	1.50	1.50
	**NZ**	2.10	2.10	2.10	2.00	2.00	2.00	1.90	1.90	1.90

**Double-leg vertical squat jump (cm)**	**SA/NZ**	50	50	45	45	45	40	40	40	40

**Single-leg vertical squat jump (cm)**	**SA/NZ**	40	40	35	35	35	35	30	30	30

**5 m sprint (s)**	**SA**	1.10	1.10	1.10	1.10	1.10	1.10	1.15	1.15	1.15
	**NZ**	1.08	1.08	1.08	1.10	1.10	1.10	1.12	1.12	1.12

**10 m sprint (s)**	**SA**	1.90	1.90	1.90	1.95	1.95	1.95	2.00	2.00	2.00
	**NZ**	1.85	1.85	1.85	1.90	1.90	1.90	1.95	1.95	1.95

**40 m sprint (s)**	**NZ**	7.30	7.30	7.30	7.35	7.35	7.35	7.40	7.40	7.40

**Fatigue index (%)**	**SA/NZ**	5–8	5–8	5	5–8	5–8	5–8	10	10	5–8

**Yo-Yo (level)**	**SA**	15.5	16.5	17.5	15.3	16.3	17.3	15.1	16.1	17.1
	**NZ**	16.1	17.5	18.5	15.5	16.5	17.5	15.1	16.1	17.1

GS, goal shooter; GA, goal attack; WA, wing attack; C, centre; WD, wing defence; GD, goal defence; GK, goal keeper; SA, South Africa; NZ, New Zealand.

## References

[1] Reid DA Vanweerd RJ Larmer PJ The inter and intra rater reliability of the Netball Movement Screening Tool J Sci Med Sport 2015 18 3 353 357 10.1016/j.jsams.2014.05.008 24930074

[2] Venter RE Fourie L Ferreira S Physical and physiological profiles of Boland netball players S Afr J Sports Med 2005 17 2 3 7 10.17159/5082

[3] Pillay T Frantz JM Injury prevalence of netball players in South Africa: The need for injury prevention S Afr J Physiother 2012 68 3 7 10 10.4102/sajp.v68i3.17

[4] Paz GA Gabbett TJ Maia MF Physical performance and positional differences among young female volleyball players J Sports Med Phys Fitness 2017 57 10 1282 1289 10.23736/S0022-4707.16.06471-9 27385546

[5] Booysen MJ Bentel D Harry K Anthropometric variables and physical fitness characteristics of male South African semi-professional footballers S Afr J Res Sport PH 2018 40 2 11 21

[6] Soh SG Soh KL Sofian OF Body fat and somatotype among Malaysian school netball players by playing performance Asia J Phys Educ Recreat 2009 15 1 56 60

[7] Fox A Spittle M Otago L Activity profiles of the Australian female netball team players during international competition: implications for training practice J Sports Sci 2013 31 14 1588 1595 10.1080/02640414.2013.792943 23672529

[8] Netball New Zealand Netball New Zealand Fitness Testing Guidelines & Protocols. Players & Umpires Auckland Netball New Zealand 2014 https://studylib.net/doc/8650080/nnz-fitness-testing-guidelines-protocols-2014 accessed 1 February 2019

[9] Taylor L Lander P Adolescent netball players normative data and physical performance profiles New Zealand Physical Educator 2018 51 1 20 24

[10] Ferreira MA Spamer EJ Biomechanical, anthropometrical and physical profile of elite university netball players and the relationship to musculoskeletal injuries S Afr J Res Sport PH 2010 32 1 57 67

[11] Baechle TR Earle RW Essentials of strength training and conditioning 3rd ed Champaign, IL Human Kinetics 2008 255 256

[12] Stewart A Marfell-Jones M International Society for Advancement of Kinanthropometry International standards for anthropometric assessment Lower Hutt, NZ International Society for Advancement of Kinanthropometry 2011

[13] Reiman MP Manske RC Functional testing in human performance: 139 tests for sport, fitness and occupational settings Champaign, IL Human Kinetics 2009 108 109

[14] Gonzalo-Skok O Serna J Rhea MR Marin PJ Relationships between functional movement tests and performance tests in young elite male basketball players Int J Sports Phys Ther 2015 10 5 628 638 26491613 PMC4595916

[15] SAS Institute Inc *SAS/STAT* 143 User’s Guide: High Performance Procedures Cary, NC SAS Institute Inc 2017 Pg nos?

[16] Taylor K-L Bonetti D Tanner R Protocols for the physiological assessment of netball players Canberra Australian Institute of Sport 2014 https://www.clearinghouseforsport.gov.au/__data/assets/file/0004/623362/Netball_National_Protocols_NP_Netball_v2.1_2014.pdf accessed 1 February 2019 Pg nos?

[17] Thomas C Ismail KT Comfort P Physical profiles of regional academy netball players J Trainol 2016 5 2 30 37 10.17338/trainology.5.2_30

[18] Thomas C Comfort P Jones PA Strength and conditioning for netball: a needs analysis and training recommendations Strength Cond J 2017 39 4 10 21 10.1519/SSC.0000000000000287

[19] Netball South Africa Pretoria: Netball South Africa 2016 http://www.netball-sa.co.za accessed 1 February 2019 Is this title correct?

[20] Thomas C Ismail KT Simpson R Physical profiles of female academy netball players by position J Strength Cond Res 2019 33 6 1601 1608 10.1519/JSC.0000000000001949 28426516

[21] CiCek G Güllü A Güllü E The effect of aerobic and core exercises on forced vital capacity Phys Cult Sport Stud Res 2018 77 41 47 10.2478/pcssr-2018-0005

